# Residual Stress Relaxation After Aging Heat Treatment in Laser Powder Bed Fusion CM247LC Superalloy Parts Quantified by Neutron-Diffraction-Validated Full-Field Eigenstrain Tomography

**DOI:** 10.1007/s40964-026-01845-y

**Published:** 2026-08-07

**Authors:** Fatih Uzun, Hector Basoalto, Tung Lik Lee, Alexander M. Korsunsky

**Affiliations:** 1 University of Oxford, Oxford, UK; 2 University of Sheffield, Sheffield, UK; 3 ISIS Neutron and Muon Source, Didcot, UK

**Keywords:** Additive manufacturing, Post-build heat treatment, Full-field reconstruction, Inverse modelling, Synchrotron X-ray diffraction, Viscoplastic creep

## Abstract

Residual stresses generated during laser powder bed fusion (LPBF) can compromise dimensional stability and in-service performance of nickel-based superalloy components, while the quantitative assessment of stress relaxation during post-build heat treatment remains challenging in three dimensions. This paper quantifies the effects of an aging treatment at 900 °C for 8 h on the residual stress and eigenstrain distributions in an LPBF-fabricated CM247LC component. Eigenstrain tomography is used to reconstruct full-field three-dimensional distributions of eigenstrain, residual elastic strain, and residual stress tensors from synchrotron X-ray diffraction measurements of a single residual elastic strain component acquired from only two orthogonal projections. Reconstruction accuracy is independently validated using neutron diffraction strain measurements. The results show substantial residual-stress relaxation after aging, including a marked reduction in the magnitude of all residual stress tensor components across the volume. Statistical analysis of the raw diffraction data demonstrates a pronounced homogenisation of internal macroscopic strains, accompanied by a corresponding decrease in reconstructed eigenstrain components toward near-zero values. Leveraging a highly streamlined two-projection tomographic protocol, these findings quantitatively reveal the three-dimensional mechanics of macroscopic (Type I) stress relaxation without the need for exhaustive experimental beamtime. The results provide critical volumetric data linking thermal exposure to the elimination of distortion-driving eigenstrain gradients. Ultimately, this work represents a significant advancement in additive manufacturing process evaluation, establishing this efficient tomographic framework as a practical, non-destructive tool for optimizing post-build heat treatments and ensuring dimensional stability in complex additively manufactured superalloys.

**Graphical abstract:**

**Figure:**
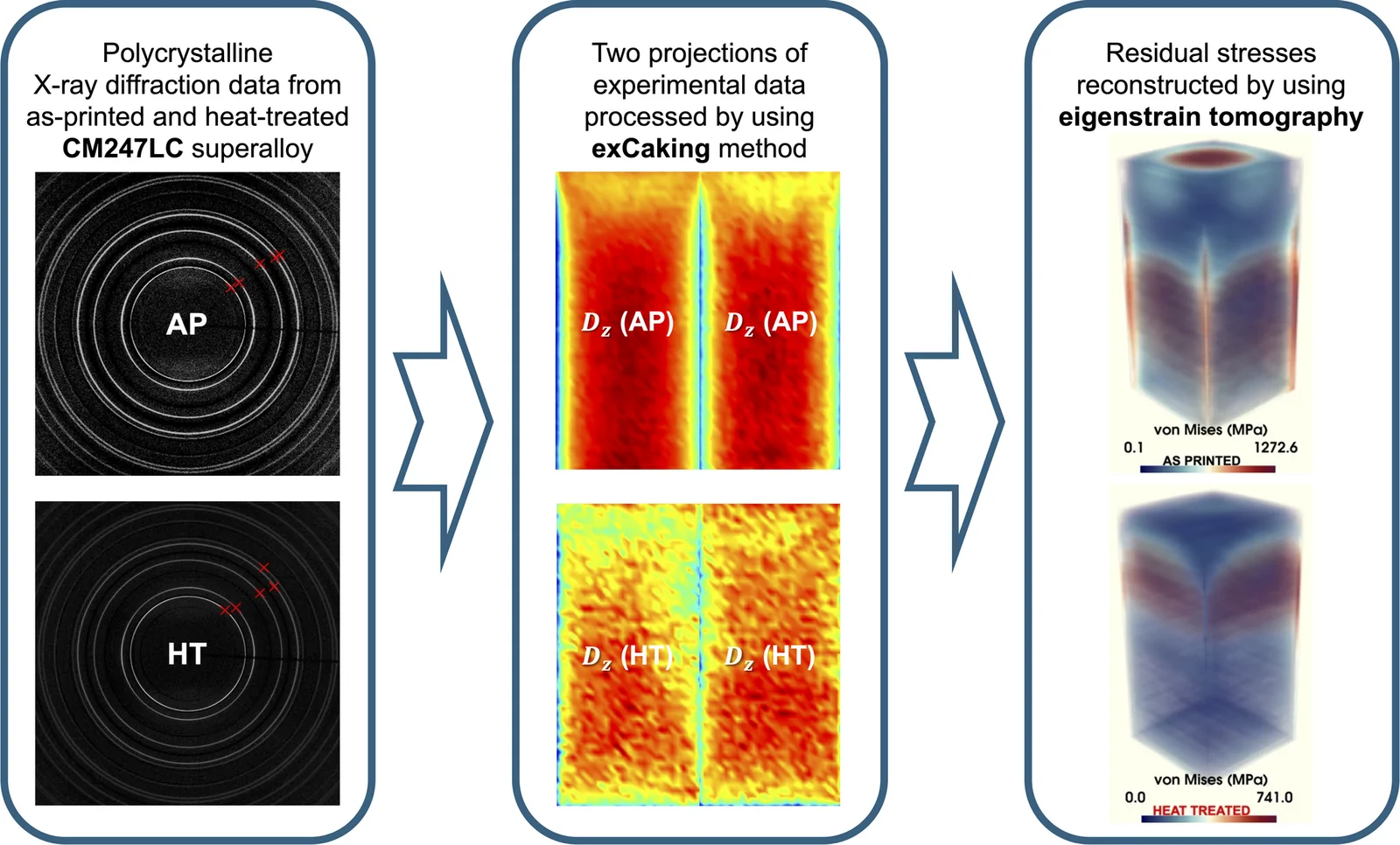

## Introduction

Nickel-based superalloys are a class of high-performance materials renowned for their exceptional mechanical properties at elevated temperatures [1, 2], including superior creep resistance [3–5], high tensile strength [6–8], and outstanding oxidation and corrosion resistance [9–11]. These characteristics render them indispensable in critical applications within demanding industries such as aerospace, where they are essential components of gas turbine engines [12–14], enabling enhanced operational efficiencies and structural integrity under extreme conditions. Laser powder bed fusion (LPBF) additive manufacturing techniques have emerged as a viable approach for fabricating complex geometries in nickel superalloys [15–17], allowing for the production of near-net-shape parts with intricate features and improved material utilisation [18–20]. CM247LC, specifically, is a precipitation-strengthened nickel-based alloy, designed with high refractory element additions to achieve optimal high-temperature performance [21–23]. This alloy exhibits a desirable combination of strength, creep resistance, and environmental durability, making it well-suited for demanding service environments [24–29]. However, the rapid solidification and complex thermal histories inherent in LPBF can result in the development of significant residual stresses that influence the structural integrity and dimensional accuracy of the fabricated components [30–32]. Throughout this work, residual stresses refer specifically to macroscopic (Type I) stresses, which represent self-equilibrating stress fields that arise from incompatible deformation gradients at the component scale and are distinct from intergranular (Type II) and atomic-scale (Type III) stress contributions that are below the resolution of the tomographic reconstruction.

The rapid and localised heating and cooling cycles inherent in LPBF processes inevitably lead to the development of complex and often unpredictable residual stress distributions within fabricated CM247LC components [33–35]. These residual stresses [36, 37], if left unaddressed, can compromise the structural integrity [38], dimensional stability [39], and fatigue performance [40, 41] of critical superalloy components [42, 43], particularly those operating in demanding environments [44, 45], making their accurate characterisation [46–49] and management essential for aerospace [50, 51] and other high-performance applications [52, 53]. Post-processing heat treatments, such as stress-relieving and aging cycles, are commonly employed to mitigate these residual stresses in nickel-based superalloys [54–56]. In particular, heat treatments play a crucial role in modifying the microstructure [57–59], relieving residual stresses and improving the mechanical properties. The effects of aging heat treatments in achieving stress relaxation are a well-documented practice in several classes of superalloys, including Inconel 718 [60] where it is used to promote the precipitation of strengthening phases, and Ti-based alloys which leads to alteration of the existing grain structures [61]. During high-temperature exposure at 900 °C, macroscopic stress relaxation in CM247LC competes with microstructural evolution where thermally activated creep mechanisms convert macroscopic elastic strains into localised permanent deformations (inelastic strains). While the total plastic strain accumulates during this process, the macroscopic incompatible eigenstrain field, acting as the source of residual stress, is resolved, leading to its substantial reduction. Also concurrent *γ*′ precipitation pins dislocations and dictates the kinetic limits of this bulk stress relief [62, 63].

Traditional methods for characterizing residual stress fields, such as hole-drilling [64–66], ultrasonic [67–70] and slitting [71–74] techniques, typically provide localised measurements limited to specific depths and locations, often disrupting the component. Furthermore, these methods primarily measure surface or near-surface strains or displacements, necessitating the use of elastic constitutive relations to infer the stress state or provide limited information about the bulk elastic state, thus lacking a complete depiction of the residual stress field in the component. Conversely, residual stress reconstruction techniques offer an attractive alternative by mapping the complete stress field using experimental data. The contour method, for example, reconstructs residual stress distributions from relaxed displacements measured after sectioning the part [75–80]. Eigenstrain (inherent strain) reconstruction [81–84], a complementary approach, employs elastic response of materials measured in terms of strain [85] or profilometry [86–89] techniques as inputs for calculations to determine the underlying eigenstrain fields, which can then be related to residual stresses via linear elastic constitutive laws with the help of principles of artificial intelligence [90, 91]. However, reconstruction techniques are commonly limited to the field and the component of experimental data [92, 93], lacking insight from three-dimensional volumes. In contrast, eigenstrain tomography [94] stands out as a powerful novel technique for full-field three-dimensional reconstruction of residual stress and eigenstrain fields. It leverages spatially resolved measurements of a single strain component obtained from techniques such as synchrotron X-ray diffraction as inputs for reconstruction, providing the complete three-dimensional eigenstrain and subsequent stress tensor distribution through robust numerical algorithms. The method’s capability to accurately reconstruct the stress state has been independently validated in studies by neutron diffraction experiments, and has proven high levels of accuracy, reliability and feasibility for experimental reconstruction purposes.

LPBF processing of nickel-based superalloys such as CM247LC produces steep thermal gradients that generate highly heterogeneous three-dimensional residual stress fields, directly affecting distortion, cracking susceptibility and the response to post-build heat treatments. While the rigorous mathematical framework and numerical validation of eigenstrain tomography have been recently established using comprehensive datasets, its application to evaluating volumetric stress changes before and after industrial heat treatments remains unexplored. Addressing this critical gap in additive manufacturing process qualification, the present study demonstrates a progressive approach by applying the previously established framework to a streamlined two-projection protocol. This allows for the volumetric tracking of stress relaxation mechanisms with significantly reduced experimental requirements. The three-dimensional relaxation of macroscopic (Type I) residual stress and eigenstrain fields is quantified in an LPBF CM247LC component subjected to an aging treatment at 900 °C for 8 h. The objective is to advance the current understanding of the internal mechanics of stress relief, specifically how thermally activated processes eliminate distortion-driving eigenstrain gradients. By providing critical volumetric data that links thermal exposure to macroscopic dimensional stability, this study establishes a highly efficient, experimentally grounded framework to optimise post-processing strategies for additively manufactured superalloys. The tomographic framework applied here was developed and numerically validated in the foundational methodological study of eigenstrain tomography [94]. The contribution of the current work is the first experimental application of that framework to quantify volumetric stress relaxation resulting from an industrial post-build heat treatment. Furthermore, by coupling the reconstruction with model-independent statistical analysis and rigorous validation against independent neutron diffraction measurements, this study demonstrates that a reduced two-projection protocol provides sufficient experimental fidelity for this purpose.

## Methodology

**Fig. 1:**
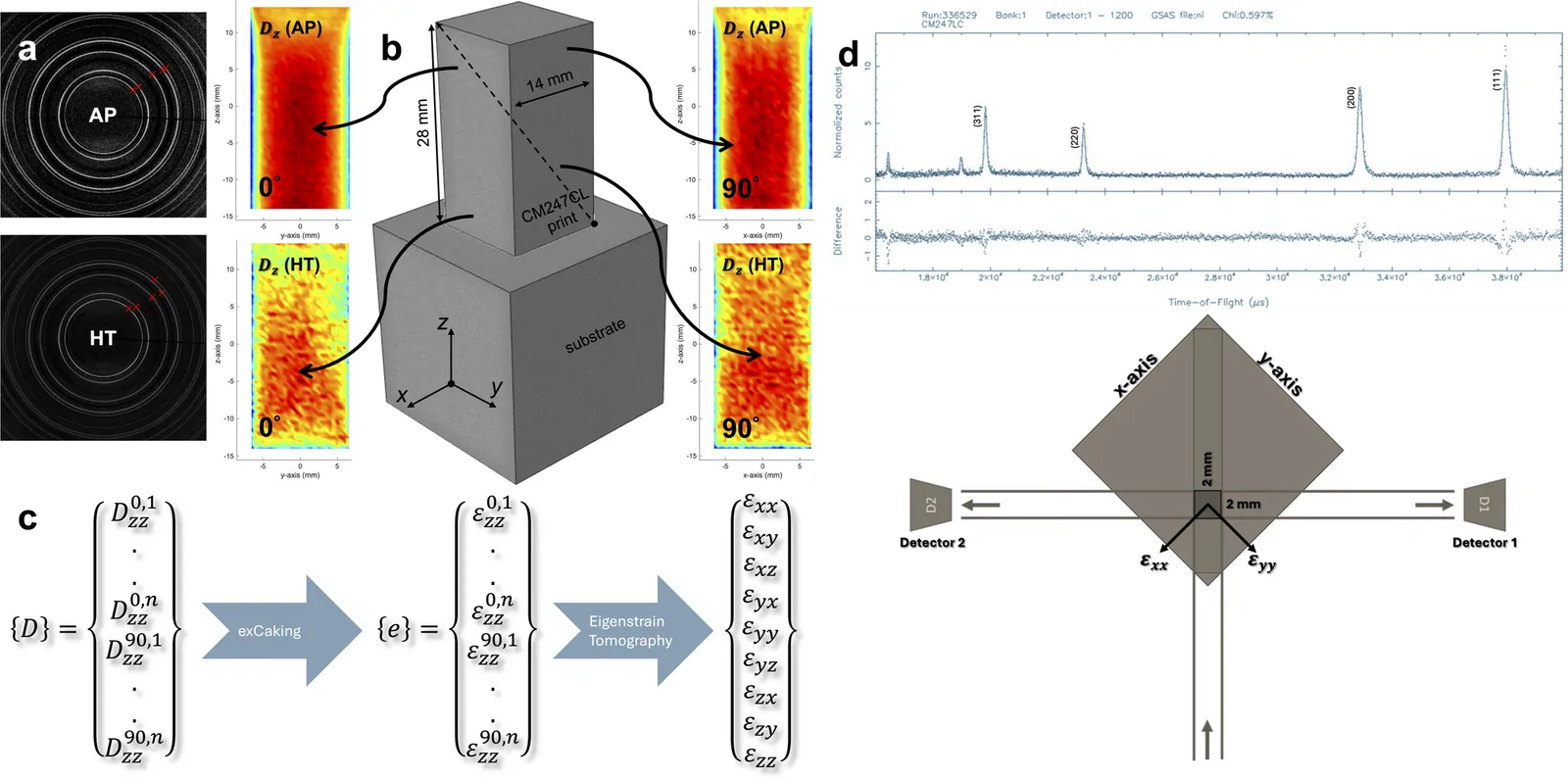
Overview of the experimental data acquisition and processing workflow. **a** Representative synchrotron X-ray diffraction patterns showing Debye-Scherrer rings from the CM247LC component. **b** Projection of radial ring distances, {*D*_*zz*_}, onto two-dimensional grids for the *xz*- and *yz*-planes, acquired via triaxial translations over orthogonal sample orientations. The raw diffraction data was processed using the exCaking method to extract precise interplanar spacings (strain) for both as-printed (AP) and heat-treated (HT) conditions. **c** The vector of distances, {*D*}, derived from 0- and 90-degree projections, calculated by the exCaking method, allows for the calculation of the *zz*-component of residual elastic strain. These fields serve as the input for eigenstrain tomography, enabling the full three-dimensional reconstruction of residual elastic strain and stress tensors. **d** Representative time-of-flight neutron diffraction spectrum along the volume diagonal, alongside a schematic of the neutron diffractometer setup used to independently quantify the *xx*- and *yy*-components of residual elastic strain

The CM247LC superalloy specimens used in this study were fabricated via LPBF, an additive manufacturing technique known to induce complex thermal gradients and associated microstructural variations, resulting in intricate and often unpredictable residual stress distributions. The specimens were fabricated from gas-atomised CM247LC powder (15–45 μm) using independently optimised LPBF process conditions that include a laser power of 200 W, scan speed of 800 mm/s, layer thickness of 30 μm, and hatch spacing of 0.1 mm. A meander scan strategy with 67° rotation between layers was employed to ensure a representative, high-density as-built state. This approach established a fundamental residual stress distribution, minimizing the influence of specific geometry-driven stress mitigation effects during the subsequent tomographic reconstruction. This approach ensured that the eigenstrain tomography process, performed independently at a separate facility, was free from potential design-related artifacts. The geometric characteristics of the printed part with dimension of 14 × 14 × 28 mm, including its overall dimensions and the position of the Cartesian coordinate system origin at the part’s midpoint, are detailed in Fig. 1.

To investigate the influence of aging heat treatment on macroscopic stress relaxation and eigenstrain changes in the LPBF-fabricated CM247LC superalloy, a controlled heat treatment regime was implemented. Specifically, the as-printed specimen was subjected to a sub-*γ*′-solvus aging treatment at 900 °C for 8 h. This temperature was selected to lie within the *γ*′ precipitation and coarsening regime of CM247LC, well below the *γ*′ solvus, which has been reported in the range of 1230–1260 °C for this alloy [57–59], so as to promote thermally activated dislocation rearrangement and viscoplastic creep-driven stress relaxation without triggering recrystallisation or grain boundary migration, processes that are suppressed below the sub-*γ*′-solvus threshold in LPBF CM247LC [58]. The aging-only protocol was adopted deliberately to isolate the macroscopic stress relaxation response in the as-printed columnar microstructure without the confounding microstructural reset, including *γ*′ dissolution, re-precipitation, and grain coarsening, introduced by a prior full solution treatment [57, 58]. At 900 °C, thermally activated creep mechanisms drive the conversion of incompatible macroscopic elastic strains into localised plastic deformation, while concurrent *γ*′ precipitation and coarsening progressively pin dislocation motion and dictate the kinetic limits of bulk stress relief [62, 63]. The soak duration of 8 h was chosen to allow sufficient thermal exposure for substantial macroscopic (Type I) stress relaxation to occur while remaining within a practically relevant single-step post-build treatment window consistent with reported aging schedules for this alloy class [23, 59]. Following the 8-hour hold, the specimen was cooled slowly inside the furnace to minimise the introduction of new thermal stresses. To characterise the effects of this heat treatment, synchrotron X-ray diffraction measurements necessary for eigenstrain tomography calculations were conducted both before and after the aging process. This pre- and post-treatment approach allows for a direct comparative quantification of the changes in eigenstrain and the corresponding residual elastic strain and residual stress tensor fields within the material as a direct result of the aging heat treatment.

To comprehensively characterise the residual stress state within the LPBF-fabricated CM247LC specimen, eigenstrain tomography [94] was employed using two projections of X-ray diffraction data to reconstruct all tensor components of residual elastic strains and subsequently, residual stresses. In this methodology, eigenstrain, following the terminology of Mura [81], denotes the incompatible inelastic strain field that acts as the source of residual stress and is distinct from the total plastic strain accumulated during deformation. This reconstruction utilises polycrystalline X-ray diffraction data acquired from two orthogonal projections of the sample. Synchrotron X-ray diffraction experiments were performed at the ID15A beamline of the European Synchrotron Radiation Facility (ESRF), using a monochromatic beam with a photon energy of 100 keV and collimated to dimensions of 500⋅500 μm. The specimen was mounted on a precision stage to enable precise positioning during data acquisition. The incident beam, after transmission through the specimen, was diffracted and captured by a two-dimensional Perkin-Elmer XRD 1621 large area detector with pixel size of 172 μm, positioned at a sample-to-detector distance of approximately 0.4 m. Triaxial translation was used to raster scan the specimen over a projection grid covering the *xz*- and *yz*-planes with a 500 μm step size. Rotation around the vertical *z*-axis was strictly employed to transition between the 0 and 90° orientations, providing the two orthogonal projections required to mathematically constrain the tomographic reconstruction. Strain analysis leveraged diffraction data by determining the radial distance from the centre of the Debye-Scherrer rings, *D*_*zz*_, using specific fundamental reflections, namely {222}, {311}, {220}, {200}, and {111}. Due to the small lattice misfit between the coherent *γ* and *γ*′ phases, their diffraction peaks overlap and thus, these reflections capture the averaged lattice strain response of both phases combined, processed using the extended caking (exCaking) method [95]. This method was chosen for its demonstrated accuracy in analyzing Debye-Scherrer rings and its command-line interface, which enables the efficient, automated processing of the large datasets generated in these tomographic scans. Relying on the mechanics principle that macroscopic (Type I) residual stresses must integrate to zero over an unconstrained volume, a pseudo-strain-free reference condition was established by averaging the corresponding diffraction ring positions along the *x*-, and *z*-axes across all measurements. Although the specimen remained attached to the substrate during measurement, the substrate reaction forces are expected to decay substantially within the scanned volume where the part height of 28 mm is large relative to the substrate thickness, and the steep LPBF stress gradients are concentrated near the build platform interface rather than distributed uniformly across the full gauge region. Consequently, the upper portion of the scanned volume approaches a state of approximate self-equilibration, and global averaging of the diffraction ring positions along the *x*- and *z*-axes provides a pragmatic reference lattice parameter in the absence of a dedicated stress-free coupon. This global baseline was then used to quantify residual elastic strains and generate the vector of strains, {*ε*}, using the vector of distances, {*D*}.

The eigenstrain tomography reconstruction computes the full six-component eigenstrain tensor aligned directly with the sample coordinate system (*x*, *y*, *z*), rather than being restricted to principal strains [94]. In this implementation, the eigenstrain field is parameterised as a linear combination of piecewise-constant basis functions, where each function represents an independent inelastic contribution within a localised subdomain of the numerical model. The reconstruction was performed using a linear elastic constitutive model, employing a Young’s modulus of 200 GPa and a Poisson’s ratio of 0.3 [23], which yield the complete three-dimensional residual elastic strain and stress tensors, explicitly including all shear components. The domain of the tomography was discretised into a 28 × 28 × 56 voxel grid, aligned with the 28 × 56 pixel projection grid used for data acquisition. Both the voxel grid and the projection grid featured a spatial resolution of 500 μm, the same as the spatial resolution of the experimental diffraction data. Specifically, the *zz*-component of residual elastic strains, obtained from two orthogonal projections of the experimental data for both as-printed (AP) and heat-treated (HT) conditions, as illustrated in Fig. 1, was used for solving the linear optimisation problem. This allowed the determination of the eigenstrain distribution in the domain of the numerical model, comprising 263,424 tetrahedral elements with an average element size of 500 μm. Consistent with the boundary conditions established in the eigenstrain tomography and tomographic eigenstrain reconstruction for this specimen geometry [94, 96], a Dirichlet boundary condition was applied to the bottom surface of the numerical model. This boundary condition enforces a fixed displacement constraint on the bottom surface, serving primarily as numerical stabilisation consistent with prior implementations rather than a fully resolved physical representation of the substrate constraint.

Residual elastic strains were mapped along the specimen using the ENGIN-X beamline at the ISIS Neutron and Muon Source with a gauge volume of 2 × 2 × 2 mm^3^. Time-of-flight neutron diffraction spectra were acquired at 15 measurement locations along the specimen volume diagonal, as illustrated in Fig. 1. Lattice parameters were extracted by Rietveld refinement, with the detectors aligned normal to the scanning axis. The resulting lattice parameters were converted to elastic strain using a stress-free reference, *d*_*0*_, obtained by averaging measurements from the four corners of the top surface. These specific locations were selected because their multiple free surfaces offer minimal mechanical constraint, providing a widely accepted macroscopic stress-free approximation for bulk additively manufactured components.

## Results and discussion

A comprehensive analysis of the impact of aging heat treatment on the eigenstrain, residual elastic strain, and residual stress states in the CM247LC superalloy is presented. The alterations in these stress-related parameters, induced by the heat treatment, are detailed through three-dimensional cross-sectional plots obtained by cutting the model along the volume diagonal plane parallel to the vertical *z*-axis. This visualisation strategy offers a holistic view of the internal spatial distributions within the core of the specimen. Furthermore, line plots depicting the variation of these quantities along the volume diagonal of the sample are provided, affording a quantitative and in-depth understanding of the stress field gradients across the material. The results presented, therefore, provide a detailed assessment of the relaxation in the residual stress and strain landscape as a consequence of the aging heat treatment process.

**Fig. 2:**
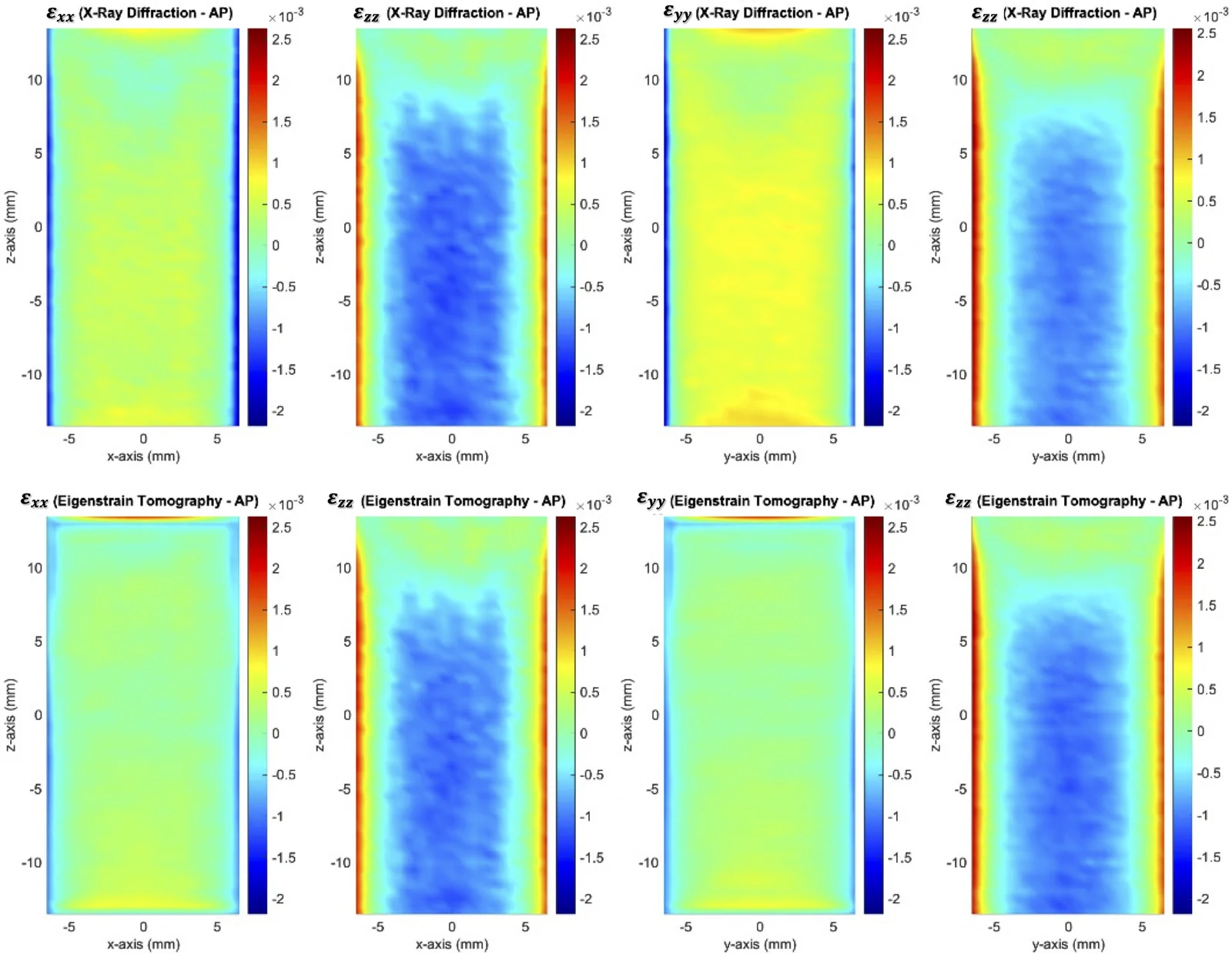
Spatial distributions of the normal components of residual elastic strains in the as-printed state. Each component is shown both as measured by synchrotron X-ray diffraction and as reconstructed using eigenstrain tomography

**Fig. 3:**
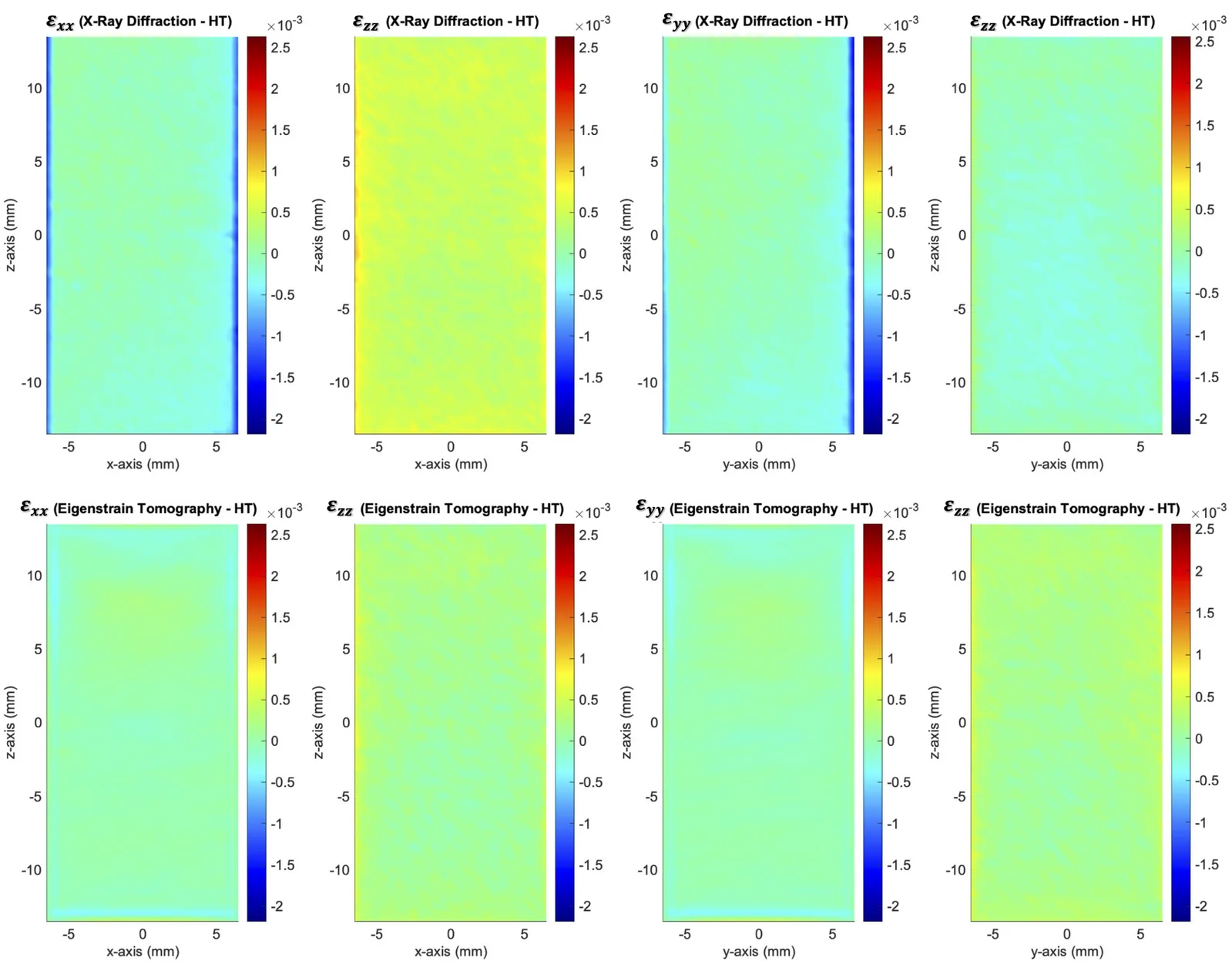
Spatial distributions of the normal components of residual elastic strains in the heat-treated state. Each component is shown both as measured by synchrotron X-ray diffraction and as reconstructed using eigenstrain tomography

The spatial distributions of the *xx*-, *yy*-, and *zz*-components of residual elastic strain, determined through synchrotron X-ray diffraction and reconstructed via eigenstrain tomography, are presented in Figs. 2 and 3. These figures display the strain components across the 14 × 28 mm projection grid, enabling a direct comparison between the as-printed and heat-treated conditions, thus illustrating the stress relaxation induced by the aging heat treatment. Moreover, the comparison between the experimentally measured strain values and the reconstructed fields confirms the physical admissibility of the calculated three-dimensional stress state prior to analyzing the material’s thermal response. Although the inverse problem relies solely on the *zz*-component as input, the solution is constrained by mechanical equilibrium and strain compatibility enforced within the finite element framework. The numerical framework enforces mechanical equilibrium through a weak-form finite element formulation and ensures total-strain compatibility by deriving the strain field from a continuous displacement field. The uniqueness of the three-dimensional tensor reconstruction from limited projections is achieved by solving the resulting system in a least-squares sense. Specifically, the use of a minimum-norm solution property, via the Moore–Penrose pseudoinverse approach, effectively selects the most physically parsimonious distribution that satisfies the experimental projections. This physical consistency is essential for accurately interpreting the volumetric relaxation of macroscopic (Type I) stresses during aging. Notably, a strong agreement between the reconstructed and measured strain distributions is observed in the as-printed state, with a root mean squared error of 9.43 × 10^− 5^ while that for the heat-treated state increases to 3.04 × 10^− 4^. This is because the absolute residual elastic strains in the aged condition approach the physical resolution limit of diffraction measurements, inherently reducing the signal-to-noise ratio. Consequently, while absolute strain deviations remain minimal, reconstructions of near-stress-free states are more sensitive to measurement noise than datasets with pronounced strain gradients.

**Fig. 4:**
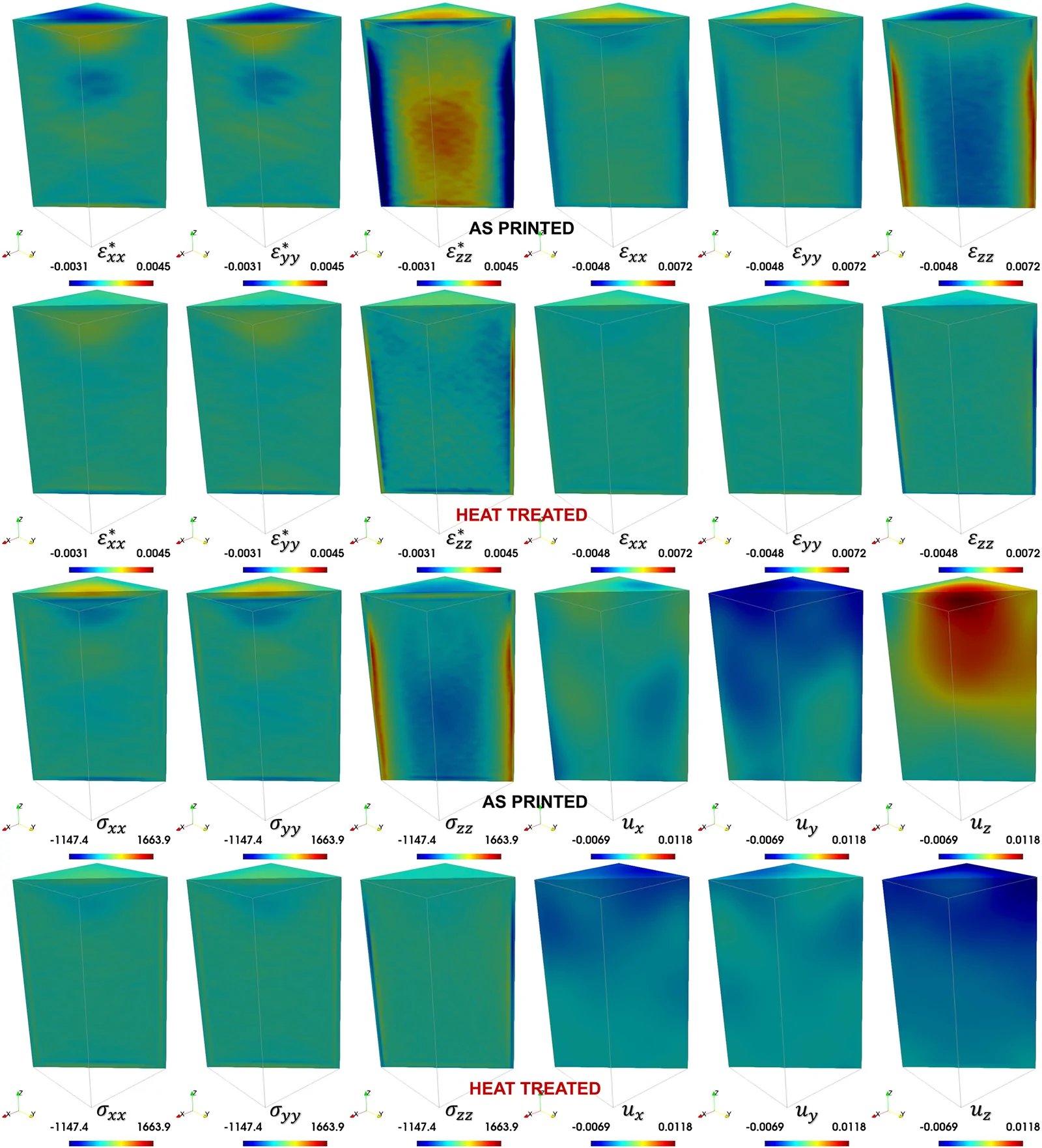
Three-dimensional distributions of all normal components of eigenstrains, ε^*^, residual elastic strains, *ε*, residual stresses, *σ* (MPa), and displacement, *u* (mm), visualised on a diagonal cross-sectional plane parallel to the vertical *z*-axis in the as-printed and heat-treated states

The spatial distributions of all normal components of stress and displacement, visualised on a vertical diagonal plane cutting through the numerical model in Fig. 4, illustrate the effect of aging heat treatment. The results show a clear reduction in the magnitude of both compressive and tensile stresses throughout the majority of the internal cross-section, with the exception of localised stress concentrations observed at regions such as the corners of the horizontal *xy*-plane. Similar to the strain distributions, the overall spatial characteristics of the stress distribution appear to be conserved, despite the reduction in stress magnitudes.

The displacement fields, which provide insight into the mechanisms of stress relaxation, exhibit significant changes in both the location and magnitude of high-displacement zones after heat treatment. These changes in displacement patterns are directly attributable to the stress relaxation process induced by aging. The quantified reduction of macroscopic eigenstrain to near-zero values provides volumetric evidence of the conversion of incompatible elastic strains into localised plastic deformation via thermally activated viscoplastic creep. This transition is confirmed by the observed redistribution of the displacement field shown in Fig. 4, which accommodates the relief of macroscopic gradients as dislocations rearrange and annihilate at 900 °C [62, 63]. Consequently, the bulk part achieves a macroscopically uniform, lower-energy state, accommodated by the shifting displacement field.

The three-dimensional distributions of all normal components of eigenstrain and residual elastic strain are visualised through cross-sectional contours along the volume diagonal in Fig. 4. These plots demonstrate the overall relaxation of both eigenstrain and residual elastic strain resulting from the aging heat treatment. The results reveal a reduction in the magnitude of both compressive and tensile strains throughout the majority of the sectioned volume, with the exception of some localised regions, such as the corners of the horizontal *xy*-plane, where strain concentrations persist. Despite the observed decrease in strain magnitudes, the overall spatial characteristics and patterns of the eigenstrain and residual elastic strain distributions appear to be largely preserved after the heat treatment, suggesting that the fundamental stress distribution is maintained, albeit at a reduced intensity. The results indicate global stress homogenisation, though persistent localised concentrations remain at the specimen corners due to boundary constraints.

**Fig. 5:**
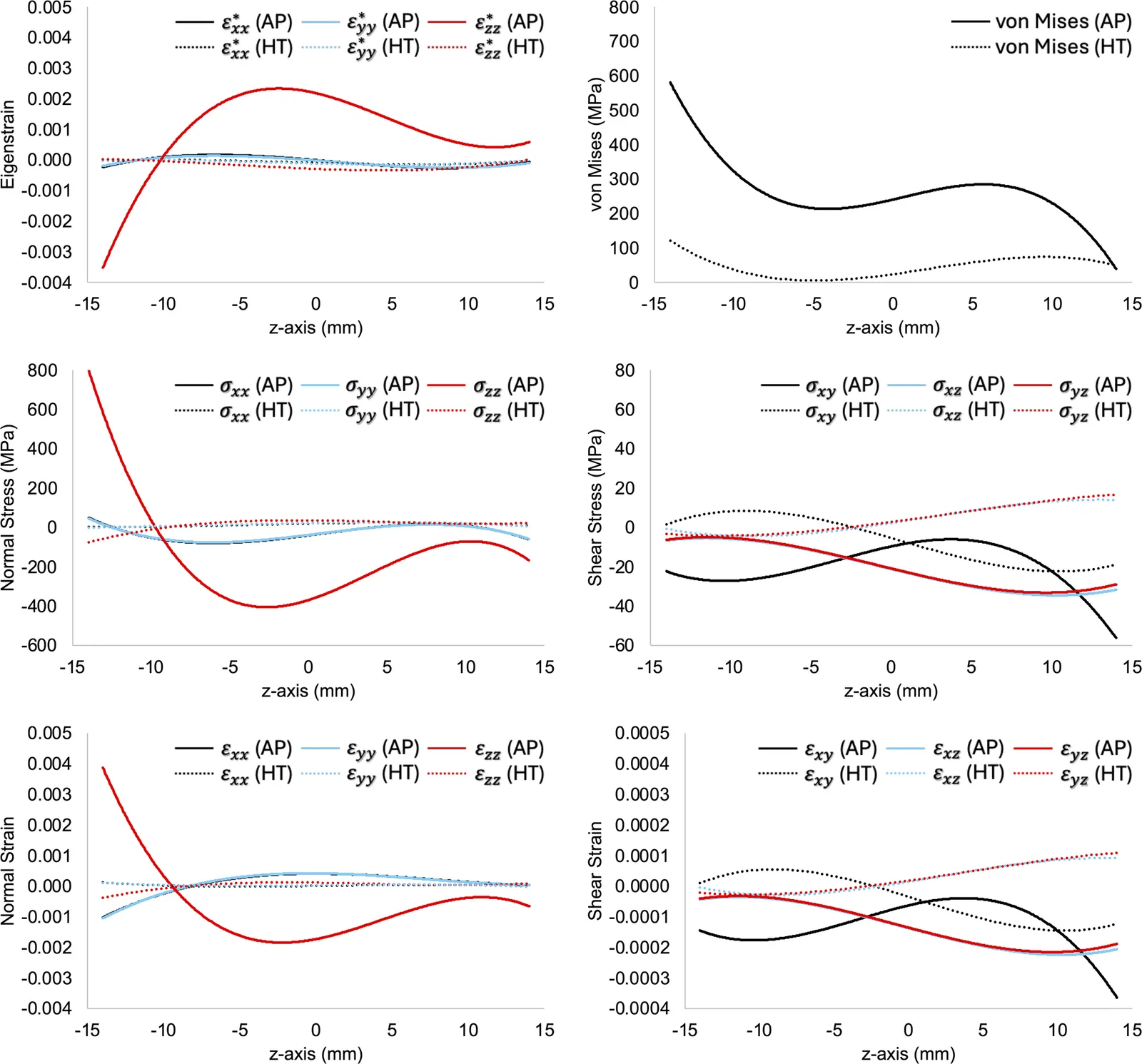
Line plots of all components of eigenstrains, ε*, residual elastic strains, *ε*, residual stresses, *σ*, along with von Mises stresses distributed along the diagonal path given in Fig. 1. The extracted data are fitted to a third-order polynomial to isolate macroscopic stress trends for direct comparison while local voxel-level noise is preserved in the contour plots

While contour plots given in Figs. 2, 3 and 4 illustrate general relaxation, localised edge hotspots can obscure broader spatial trends. To clarify these macroscopic (Type I) variations, data was extracted along the volume diagonal and fitted to a third-order polynomial in Fig. 5, effectively filtering out voxel-level noise. These profiles clearly show that aging drives all eigenstrain components toward near-zero values. Although the Moore–Penrose pseudoinverse naturally favors low-magnitude (minimum-norm) solutions in low-signal regimes, independent neutron diffraction validation of the as-printed state confirms that this calculated reduction reflects genuine physical stress relaxation rather than a mathematical bias. Consequently, the corresponding residual elastic strain and stress components also exhibit a marked decrease, converging toward zero, most notably at the specimen corners.

In the as-printed state, shear stress components are approximately an order of magnitude smaller than normal stresses, reflecting the near-orthotropic symmetry induced by the LPBF meander scan strategy with a 67° interlayer rotation. Following aging, the proportional reduction of these shear components confirms global, rather than directionally selective, stress relief. The small, non-zero shear stresses persisting post-treatment stem from residual geometric asymmetries in the eigenstrain field, consistent with substrate boundary constraints, rather than numerical reconstruction artifacts.

Initially, macroscopic residual stresses exhibit high magnitudes at the specimen corners. These localised concentrations are sustained by severe multi-axial constraints and cyclic work hardening inherent to LPBF thermal gradients, aligning with previously reported as-printed CM247LC stress levels [23, 59]. Following heat treatment, these extreme stress concentrations are nearly eliminated. The von Mises stress profiles comprehensively capture this relaxation, demonstrating a globally reduced and homogenised stress state. Furthermore, the line plots confirm that all residual stress and strain components, along with their sharp spatial gradients, are flattened. This statistical narrowing and gradient elimination quantitatively prove that high-temperature exposure resolves internal incompatibilities, effectively exhausting the stored elastic energy responsible for driving macroscopic component distortion [62, 63].

**Fig. 6:**
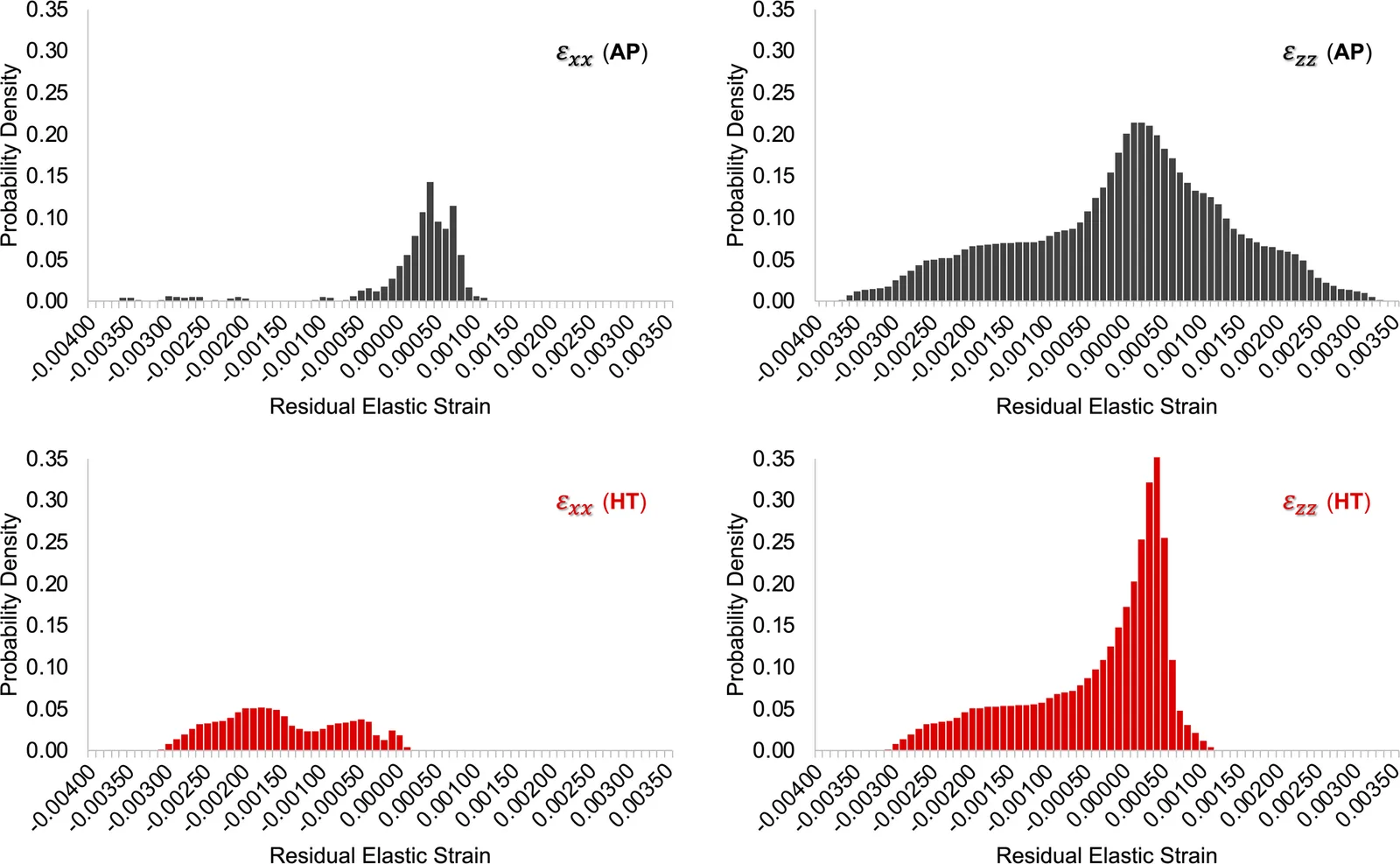
Probability density functions of the experimentally measured *xx*- and *zz*-components of residual elastic strains derived directly from the raw synchrotron X-ray diffraction 0° and 90° projections. The histograms illustrate the statistical distribution of macroscopic strains across the specimen in the as-printed (AP) and heat-treated (HT) conditions. The transition from broad, highly dispersed strain profiles in the AP state to sharply peaked distributions centered near zero in the HT state provides direct, model-independent experimental evidence of bulk stress homogenisation

Furthermore, the fidelity of the current reconstruction, derived from a reduced experimental dataset of two orthogonal projections, was assessed in the context of the eigenstrain tomography framework established in previous methodological work [94]. It should be noted that this study applies the two-projection protocol to a CM247LC specimen and the six-projection protocol to an AlSi7Mg specimen, meaning that a direct benchmark comparison between projection densities for an identical CM247LC specimen is not available from that work. Instead, validation of the two-projection protocol in the present study rests on the independent neutron diffraction measurements, which show strong agreement in the *xx*- and *yy*-components that were not used as input to the reconstruction. This confirms that robust three-dimensional eigenstrain tomography reconstructions can be achieved with significantly reduced synchrotron beamtime requirements without compromising the physical admissibility of the resulting tensor fields.

To quantify macroscopic strain homogenisation directly from raw experimental data, independently of both the numerical reconstruction and any spatial registration between pre- and post-treatment datasets, a statistical analysis of the probability density distributions of the synchrotron diffraction strain fields was conducted as shown in Fig. 6. Because ex-situ heat treatments preclude exact spatial point-to-point tracking due to minor remounting alignments, plotting the probability density function of the experimental strain fields provides a robust, global metric for relaxation. Figure 6 presents the probability density distributions of the *xx*- and *zz*-components of residual elastic strains derived directly from 0° and 90° two-dimensional projection data, respectively. In the as-printed state, the probability density functions are notably broad and widely dispersed, reflecting the highly heterogeneous, steep strain gradients inherently induced by the LPBF thermal cycles. Following heat treatment, the experimental distributions exhibit a dramatic statistical narrowing, with the vast majority of the measured data points converging into a sharp peak centered near zero. This distinct statistical shift directly quantifies the bulk homogenisation of the internal stress state, providing model-independent, empirical evidence that the component has transitioned into a macroscopically uniform, lower-energy state.

**Fig. 7:**
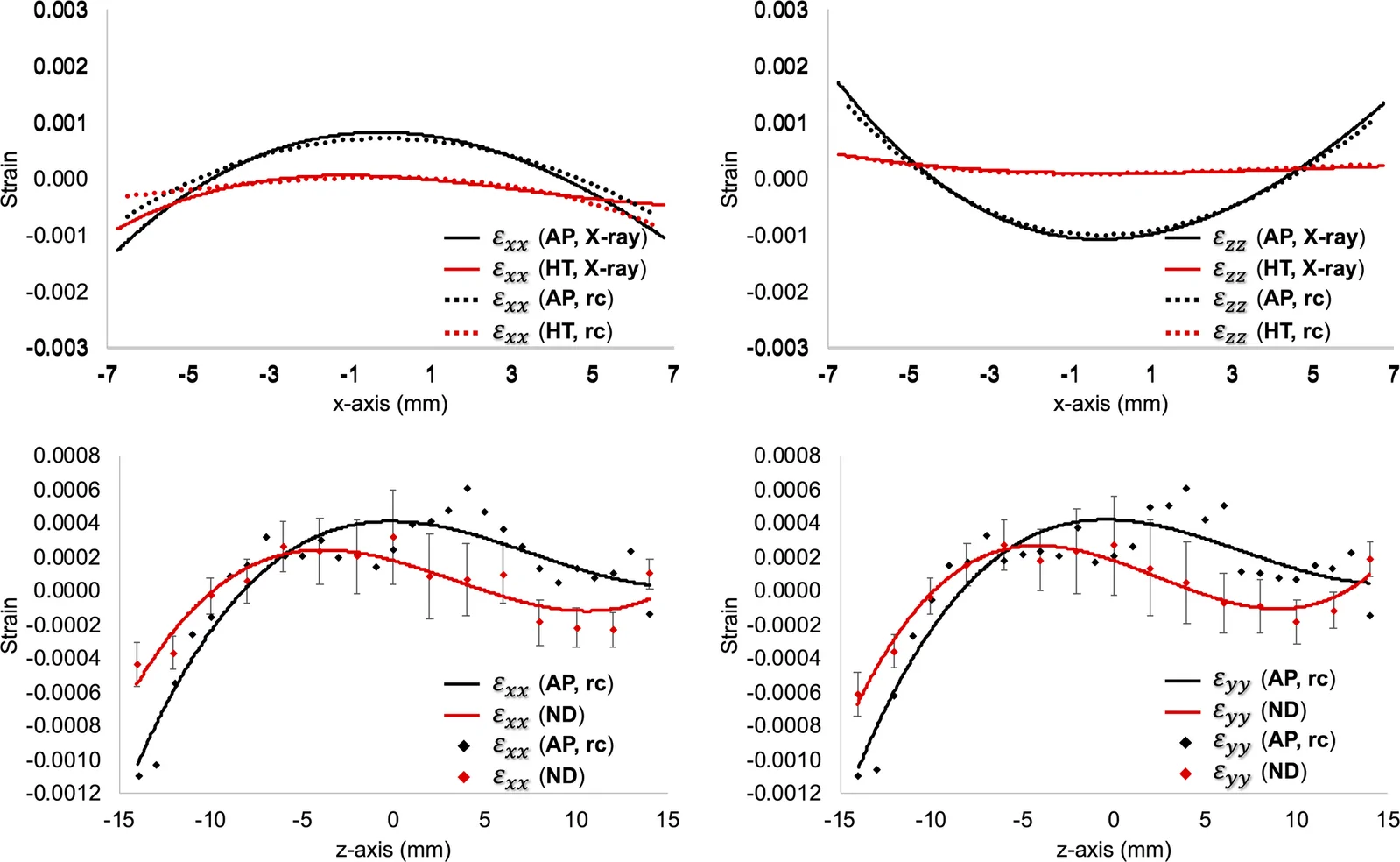
Quantitative validation by macroscopic trend line analysis of the reconstructed residual elastic strain fields. The top row shows X-ray diffraction (X-ray) and reconstruction (rc) profiles of the *xx*- and *zz*-components of residual elastic strains plotted along the central *x*-axis, directly comparing the as-printed (AP) and heat-treated (HT) states to illustrate the elimination of macroscopic strain gradients. Bottom row shows experimental validation comparing the reconstructed (rc) *xx*- and *yy*-components of residual elastic strains in the AP state against independent experimental neutron diffraction (ND) measurements obtained along the volume diagonal of the specimen. All datasets are fitted to a third-order polynomial to isolate underlying macroscopic (Type I) strain gradients from local, voxel-level microstructural variations

Having established bulk homogenisation empirically in Fig. 6, the physical admissibility of the three-dimensional tensor reconstruction is assessed here through two independent comparisons. The first compares the reconstructed fields directly against the synchrotron input data along the central *x*-axis to confirm fidelity of the reconstruction to its input. The second, and more stringent, validation compares the reconstructed *xx*- and *yy*-components against neutron diffraction measurements. Since the tomography algorithm uses only the *zz*-component as input, agreement in the orthogonal components constitutes a genuinely independent cross-validation of the full tensor reconstruction rather than a self-consistency check. To isolate the underlying macroscopic (Type I) strain trends from localised, voxel-level microstructural noise, the extracted data sets were fitted to a third-order polynomial. The top row of Fig. 7 provides a direct comparison between the experimentally measured X-ray diffraction data and the reconstructed fields for the *xx*- and *zz*-components, collected from all data points over the projection grid, along the central *x*-axis. The strong overlap between the experimental data and the reconstructed fits in both the as-printed and heat-treated states confirms that the numerical framework faithfully preserves the spatial characteristics and magnitudes of the raw projections without introducing spurious artifacts. Furthermore, the profiles clearly illustrate the flattening of steep strain gradients post-heat treatment, quantitatively confirming macroscopic relaxation.

The physical admissibility of the three-dimensional tensor reconstruction was independently validated using strain measurements acquired from the ENGIN-X neutron beamline. The bottom row of Fig. 7 illustrates the comparison between the reconstructed *xx*- and *yy*-components and the corresponding raw data points obtained via neutron diffraction along the volume diagonal of the as-printed specimen. It is critical to note that the tomography algorithm utilised only the *zz*-component of the X-ray strain as the input field and therefore, the strong agreement observed in the orthogonal *xx*- and *yy*-components serves as a robust cross-validation of the methodology. The reconstructed profiles accurately reproduce the experimental macroscopic strain gradients, capturing the spatial transition from compressive states at the specimen corners to tensile states at the core. While absolute strain differences are observed at certain discrete locations, these minor discrepancies are physically expected where the neutron measurements inherently average strains over a much larger 2 × 2 × 2 mm^3^ gauge volume compared to the fine voxel resolution of the X-ray reconstruction, effectively smoothing out sharp local gradients. Additionally, relying on macroscopic corner measurements for the neutron stress-free reference likely introduces a minor systematic shift, as residual constraints often remain even at the extremities of bulk additive manufacturing parts. Ultimately, this comprehensive, multi-modal correspondence confirms the capability of the technique to accurately resolve the full three-dimensional strain tensor from limited projections, establishing high confidence in the evaluated volumetric stress relaxation.

## Conclusion

A three-dimensional assessment of aging-induced relaxation of macroscopic (Type I) residual stress and eigenstrain in an LPBF-fabricated CM247LC superalloy component has been presented. By leveraging a highly efficient two-projection tomographic protocol established for eigenstrain tomography, the volumetric relaxation of the complete stress tensor was successfully quantified before and after an aging heat treatment at 900 °C for 8 h. By applying this advanced tomographic framework, this study directly quantifies the internal mechanics of stress relief in complex additive architectures, revealing how elevated thermal exposure annihilates the fundamental eigenstrain source terms responsible for component distortion.

Aging produced pronounced relaxation and partial homogenisation of the macroscopic (Type I) residual stress and strain fields at the component scale, evidenced by a clear reduction in the magnitude of residual stress components throughout the volume and by diminished spatial gradients. The reconstructions further indicate spatially non-uniform relaxation, with localised regions exhibiting stronger stress reduction, consistent with progression toward a more uniform internal elastic-energy state after heat treatment. In parallel, incompatible macroscopic eigenstrain components decreased substantially toward near-zero values across the component, signalling the resolution of the internal misfits that drive Type I stresses. Reconstructed three-dimensional fields provide volumetric evidence of the physical conversion of incompatible elastic strains into localised plastic deformation via high-temperature viscoplastic creep mechanisms. These results establish macroscopic eigenstrain as a direct quantitative measure of viscoplastic relaxation kinetics and the concurrent homogenisation of Type I residual fields in LPBF superalloys.

The physical admissibility of the reconstructed stress fields was independently validated by neutron diffraction measurements at the ENGIN-X beamline, which showed strong agreement with the reconstructed *xx*- and *yy*-components of residual elastic strain that were not used as input to the reconstruction. This agreement constitutes genuine cross-validation of the full tensor reconstruction rather than a self-consistency check, and confirms that the two-projection protocol produces physically admissible three-dimensional stress fields for this CM247LC specimen and heat treatment condition. Statistical analysis of the raw synchrotron diffraction strain distributions provides additional model-independent evidence of bulk stress homogenisation, with the probability density functions transitioning from broad, widely dispersed profiles in the as-printed state to sharply peaked distributions centered near zero following heat treatment.

From a practical additive manufacturing perspective, this highly efficient two-projection framework represents a meaningful progression in process qualification and optimisation. It enables rapid, non-destructive three-dimensional mapping of residual stress evolution during post-processing, providing experimentally grounded tensor fields essential for calibrating and validating predictive simulations of part distortion. While near-stress-free conditions present lower strain gradients that can limit the information available for inverse reconstruction, a limitation that can in principle be mitigated by additional projections or complementary constraints, the streamlined method proves highly effective for assessing primary stress relief. Extending this approach to more complex additive manufacturing geometries, and quantifying the fidelity gains achievable with additional projection angles on identical CM247LC specimens, represent important directions for future work. Integrating the reconstructed fields into process–structure–property optimisation workflows will further accelerate the development of geometry-aware residual stress management strategies for high-performance additively manufactured applications.

## Data Availability

The raw synchrotron X-ray diffraction data supporting this study will become openly available in the ESRF data portal in 2026 (https://doi.org/10.15151/ESRF-ES-1302902268). Complete volumetric reconstruction datasets, including eigenstrain, residual stress, and strain fields in .vtu format, are openly available via Mendeley Data (https://doi.org/10.17632/mtx63689t4.1).
